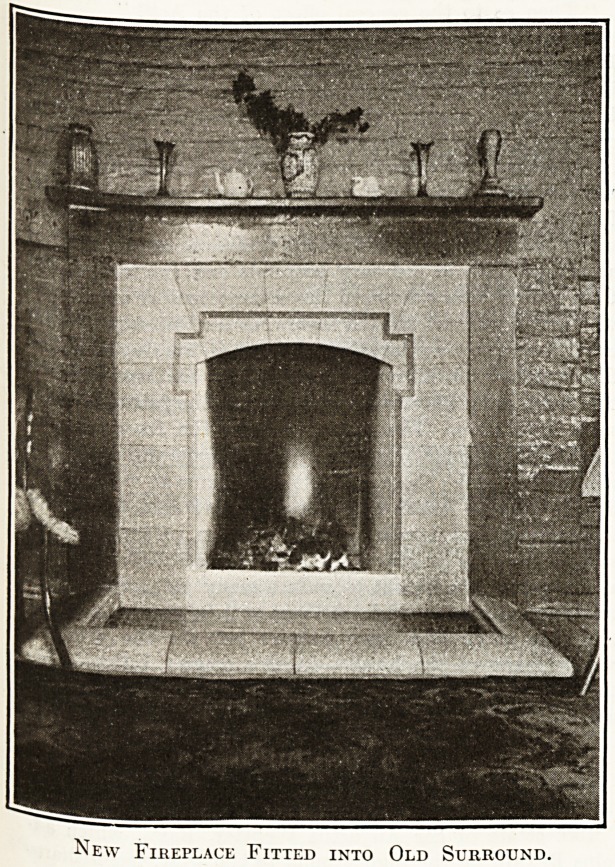# Ward Fireplaces: A New Design

**Published:** 1912-08-10

**Authors:** 


### PRACTICAL POINTS.

(Criticism and Suggestions Invited.)

### Ward Fireplaces: A New Design.

In Mid-Victorian days Miss Florence Nightingale claimed, in her *Hospital Notes*, that natural ventilation and open, radiating fireplaces are the only suitable means of renewing and warming the air of the ward.

With her claim the most eminent of our present hospital authorities are in unanimous accord; but, instead of the old-fashioned iron Victorian grate, the popular trend is towards a slow-combustion fire in a fireclay basin. It is admitted that fireclay is the best of heat absorbers, and its

New Fireplace Fitted into Old Surround.

use in the fireplace ensures that, even when the fire is low, there is always a certain amount of radiation going on. The illustration shows one of eight new fireplaces fitted into old surrounds at the Infirmary for the Guardians of the Poor of St. George's-in-the-East, London. The cost of the eight new stoves, hearths, and kerbs, fixed complete, was £40: the work was executed by Messrs. Doulton and Co.

We are indebted to the Resident Medical Superintendent, Dr. Bowlan, for the photograph from which our illustration is taken. Messrs. Doulton also show two other designs for hospital or school fireplaces. It is claimed for these that they are eminently suitable for hospitals or other institutions where hard wear and simplicity of form are essential. The three types are specially made in biscuit-coloured Carrara ware, a highly vitrified artificial stone of pleasing appearance. The cost of fireplaces fixed complete with hearth and kerbs is stated to be from £3, depending on the size, the number ordered, and the distance from London.

## Figures and Tables

**Figure f1:**